# Case study of chemical and enzymatic degumming processes in soybean oil production at an industrial plant

**DOI:** 10.1038/s41598-024-53865-9

**Published:** 2024-02-19

**Authors:** Maged Khamies, Mohamed Hagar, Taher S. E. Kassem, Amira Hossam Eldin Moustafa

**Affiliations:** 1 Chemistry Department, Faculty of Science, Alexandria University, P.O. 426 Ibrahemia, Alexandria, 21321 Egypt; 2 Faculty of Advanced Basic Sciences, Alamein International University, Alamein City, Matrouh Governorate Egypt

**Keywords:** Soybean oil, Industrial plant, Enzymatic degumming, Phospholipase, Economic impact, Health care, Chemistry

## Abstract

The vegetable oil degumming process plays a critical role in refining edible oil. Phospholipids (*PL*) removal from crude extracted soybean oil (*SBO*) by the enzymatic degumming process has been investigated in this work. Enzymatic degumming of extracted *SBO* with microbial phospholipase *A1 PLA-1* Quara LowP and Lecitase Ultra enzymes have also been studied comparatively. The main novelty of our work is the use of the enzymatic degumming process on an industrial scale (600 tons a day). Many parameters have been discussed to understand in detail the factors affecting oil losses during the degumming process. The factors such as chemical conditioning (*CC*) by phosphoric acid 85%, the enzyme dosage mg/kg (feedstock dependent), the enzymatic degumming reaction time, and the characteristics of the plant-processed *SBO* have been discussed in detail. As a main point, the degummed oil with a phosphorus content of < 10 mg/kg increases yield. Quara LowP and Lecitase Ultra enzymes are not specific for certain phospholipids PL; however, the conversion rate depends on the *SBO* phospholipid composition. After 4 h, over 99% of Phospholipids were degraded to their lysophospholipid *LPL* (lysolecithin). The results showed a significant effect of operating parameters and characteristics of different origins of *SBO*, fatty acids *FFA* content, Phosphorus content and total divalent metals (Calcium *Ca*, Magnesium *Mg* and Iron *Fe* mg/kg) content on the oil loss. The benefit of using enzymatic degumming of vegetable oils rather than traditional chemical refining is that the enzymatic degumming process reduces total oil loss. This decrease is known as enzymatic yield. The enzymatic degumming also decreases wastewater and used chemicals and running costs; moreover, it enables physical refining by lowering the residue phosphorus to < 10 mg/kg.

## Introduction

Soybean oil *SBO* is a vegetable oil extracted from soybean seeds. It is one of the most widely consumed cooking oils and the second most consumed vegetable oil^1^. As a drying oil, processed *SBO* is also used as a base for printing inks and oil paints. Soybean oil was produced worldwide, constituting about half of edible vegetable oil and thirty percent of all fats and oils produced, including animal fats and oils derived from tropical plants^2^. Refining *SBO* means removing undesirable impurities that decrease the quality and the shelf-life time, such as taste, flavor, and appearance. The Phospholipids PL are the essential impurities that affect the quality of edible oil^3^**.** The *PL* are esters of glycerol, fatty acids, phosphoric acid, and other alcohols. *PL* are divided into hydratable *HPL* and non-hydratable phospholipids NHP^4^. *HPL* is removed by water, but NHP is removed by acid degumming steps^5,6^.

The *SBO* refining process operates with two types: chemical and physical refining. Physical refining is the most favored due to its environmental and cost benefits^7^. Physical and chemical refining comes from the process technology that removes the *FFA*-free fatty acids responsible for the oil's acidity. Physical refining is a process distillation of the *FFA* in the final deodorization refining step compared to the boiling point of the triglyceride oil. In chemical refining, an alkaline solution is used to neutralize the *FFA*. The chemical refining technique has been used for many centuries. The chemical refining makes saponification by neutralizing *FFA* with alkali as a caustic soda *NaOH* and diluting the resulting soaps in a water phase. Centrifuge separators remove these soaps in batches or during continuous processing. The resulting neutralized oils are subsequently bleached and deodorized. This chemical refining is the default method for refining all crude oils, including oils of low quality.

In addition to the removal of *FFA*, impurities of other materials are also removed as *PL*, oxidized products, metal ions (e.g., calcium, magnesium, iron and copper. etc.), color pigments (e.g., Chlorophyll and Carotenes), and insoluble impurities (e.g., meal fines). Removing oxidizing materials and pigments is an advantage of chemical refining rather than physical refining. The chemical refining loss is higher than the other because the caustic soda and soap act as emulsifying agents which escape neutral oil in soap stock during centrifuge separation^8^. A new physical degumming technique, known as an enzymatic degumming method, is established. The porcine pancreas phospholipase-A1 *PLA 1* commercial names are Quara LowP and Lecitase Ultra, stable enzymes available from industry10; they were used to convert the *NHP* to its hydratable form^9^. Such a process was developed to increase the yield rather than a chemical refining technique. The reduction of emulsification properties of the *PL* could be achieved chemically or enzymatically by separating the polar and nonpolar parts. However, enzymatic reactions are selective rather than chemical ones, and this will decrease the loss%^11^**.**

**Figure 1:**
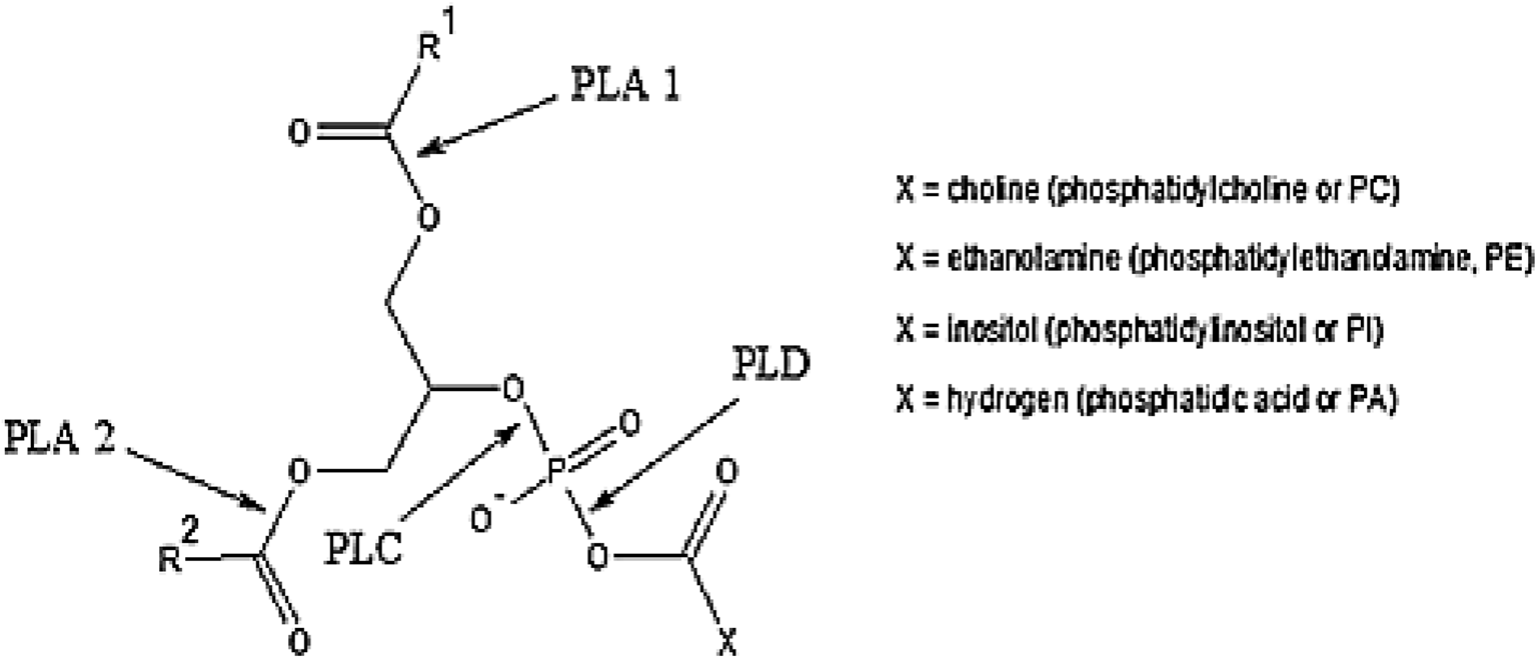
Types of lipase phospholipase enzyme reaction cites on phospholipid molecule.

Phospholipase enzymes *PLA 1, PLA 2*, Phospholipase-C *PLC*, and phospholipase-D *PLD* with a Phospholipid cleave triacylglycerol to the fatty acids in the presence of water^12^. There are three types of phospholipases: the first type is *PLA 1* and *PLA 2* enzymes specific for the free fatty acids. The second type is the enzyme *PLC*, which cleavages the phosphate group from the glyceryl part. The third is PLD, which breaks the phosphate and the non-glyceryl part^13^. Figure 1 illustrates Types of Lipase Phospholipase enzyme reactions cites on phospholipid molecules.

Enzymatic degumming and chemical refining processes in plant-scale trials were performed on the same *SBO* of different oilseed origins with a microbial phospholipase *A1* Quara LowP and Lecitase Ultra enzymes. The enzymatic degumming with Quara LowP and Lecitase Ultra enables full vegetable oil degumming, increasing oil yield. Quara LowP and Lecitase Ultra enzymes effectively release more neutral oil and *FFA* to help gain oil yield and to consistently meet the benchmark end-product specifications for phosphorous levels content < 10 mg/kg without adjusting pH and temperature. The result is lower operating costs. The Quara LowP and Lecitase Ultra enzymes have no specificity for a certain *PL*, but the conversion rate depends on the *PL* composition of the crude oil in each origin. Enzymatic degumming with Quara LowP and Lecitase Ultra releases lower neutral oil in separated gums. That's because strong activity on *PL* hydrolyzes most of them, increasing the oil yield and profitability.

These Quara LowP and Lecitase Ultra enzymes break down the emulsion in the separation stage in a gum’s separation centrifuge. It means that the removal of phosphorous is easier^9^. The Quara LowP and Lecitase Ultra also reduce the need for acid and caustic soda in the degumming process rather than chemical refining^14^. Quara LowP and Lecitase Ultra enzymatic degumming reduce the formation of low-value by-products such as gums. The economic impact is measured in terms of the increment of degummed oil yield obtained due to enzymatic reaction rather than the chemical refining process. *LPL* is a valuable feed ingredient as a feed additive^15^. The enzymatic degumming reduced environmental impact. There is no need to make treatment of washing water as in chemical refining because there are no soaps produced, the higher oil yields reduce the carbon dioxide footprint of the production, and the lower energy costs also reduce the impact of the process on the environment^16^^.^

## Experimental section

### Chemicals

#### Soybean vegetable oils

Different origins of *SBO* were obtained by solvent extraction technique using hexane as a solvent from the industrial Alex. Co. plant (Alexandria, Egypt). The specification of the different origins of *SBO* is reported in Table 1, which shows the hydrolysis phospholipids required for efficient degumming of vegetable oil.

**Table 1:** Comparative analysis of quality elements in soybean oil from different countries.

Quality elements	American SBO	Argentinian SBO	Ukrainian SBO	Uruguay SBO
FFA (as % C18:1)	0.65 ± 0.1	0.79 ± 0.1	0.93 ± 0.1	1.4 ± 0.1
P mg/kg	878 ± 15	822 ± 9	799 ± 8	762.4 ± 5
Ca mg/kg	111 ± 9	128 ± 8	134 ± 6	182 ± 7
Mg mg/kg	83 ± 3	88 ± 3	95 ± 2	123 ± 5
Fe mg/kg	2.2 ± 0.8	1.7 ± 0.5	3.2 ± 0.3	3.88 ± 0.3

#### Lecitase ultra enzymes

Lecitase Ultra product is a chimera produced by the fusion of the lipase genes from Thermocycles lanuginosus and the phospholipase *A1* from Fusarium exospore. The enzyme was first designed for the enzymatic degumming of oils. The working pH ranges from 4.5 to 5.5, and the operating temperature is 55–60 °C.

#### Quara LowP enzyme

The phospholipase enzyme *PLA 1* (Quara LowP) is provided by Novozymes A/S (Bagsvaerd, Denmark). Quara LowP is a liquid phospholipase product with high activity in Quara LowP product contains a protein-engineered carboxylic ester hydrolase (EC 3.1.1.3) produced by submerged fermentation of a genetically modified Aspergillus Niger microorganism. The enzymes hydrolyze the phospholipids at positions 1 and 2-position as the main reaction sites^17^.

Special care must be taken with the pH of the enzyme solution. The pure enzyme is stabilized in a buffer of pH = 4^18^. If mixed with the water before the addition to the oil stream, the enzyme and water must be added separately, and the addition should be as near the shear mixer as possible. The working pH ranges from 3.8 to 4.2, and the operating temperature is 70–75 °C^9^.

According to the enzyme application sheet provided by the Novozymes A/S (Bagsvaerd, Denmark), the denaturation of the enzyme is impacted by the pH values, Supplementary data Fig. S3.

#### Plant processing supporting materials

Phosphoric Acid 85% had been purchased from Food Chem. International Corporation (Shanghai, China), Caustic Soda 48% concentration commercial grade was provided from Misr Chemical Industries Co. (Alexandria, Egypt). The process water from the reverse osmosis unit is used in Chemical or enzymatic refining city water was softened to (1–1.5 mg/L total hardness as *CaCO*_*3*_).

#### PH measurements of gums

The weight of 30.0 ± 0.05 g of *SBO* gums sample was introduced in a 100 ml blue cap bottle, then 10 ml freshly prepared 1% *KCl* solution was added. The sample at 70 °C had been kept in a heating cabinet for 1 h. The samples were mixed using IKA ULTRA-TURRAX laboratory mixer at full speed (24,000 rpm) for 30 s or with a magnetic stirrer at 1000 rpm for 5minuts; the samples were warmed at 70 °C in a heating cabinet for 1h for the phases to separate. Take out the water phase with a pipette and transfer it into a 15 ml plastic tube. After the sample cooled to room temperature, measure the pH with a calibrated pH-meter^19^; all the pH measurements were performed in triplicate. The pH values of *SBO* gums were measured by a digital pH meter in the plant QC (Quality Control) laboratory^20^. The device electrode is HACH IntelliCAL PHC201 and was calibrated with HACH commercial standard buffer solutions of pH 4.01, 7, and 10.01.

#### Elements measurements

Elements analysis (*P, Ca, Mg* and *Fe*) of *SBO* and end processed oil measured in the plant *QC* laboratory by the PerkinElmer Optima™ 8000 *ICP-OES* instrument (U.S.A). The measurements of the PL content in *SBO* were calculated as a function of P oil according to the American Oil Chemists' Society (AOCS) Official Method "Ca 20-99"^21^.

According to the *AOCS* Official Method *Ca* 17-0122, the *Ca* and *Mg* metals were measured. The detection of *Fe* concentration was carried out according to the *AOCS* Official Method "*Ca* 15-75"^23^. The analysis parameters are shown in Table 2.

**Table 2:** Instrumental parameters for the analysis of *SBO* using the PerkinElmer Optima 8000 ICP.

Parameter	Setting
Ignition mode	Organics
RF power (Watts)	1500
Plasma gas (L/min)	10
Auxiliary gas (L/min)	0.8
Nebulizer gas (L/min)	0.35
Plasma view	Radial
Viewing distance (mm)	15
Sample pump rate (mL/min)	2
Auto integration	0.5 s minimum. * − 5.0 s maximum. Ca, Mg, Fe at 0.5 s minimum. − 10.0 s maximum. P
Replicates	3
Background correction	2-point
Internal standard	Cobalt
Calibration model	Linear through zero

#### Free fatty acids measurements

*FFA* of *SBO* is determined by titration with a standard alkali, Sodium Hydroxide *NaOH*, of specific normality *N* according to the *AOCS* official method *Ca* 5a‐40, and is expressed as oleic Acid (C18:1)^24^ by Replicates 3 times for each test.

A 7.00 ± 0.05 (g) of *SBO* was weighed in the Erlenmeyer flask. Then about 25 ± 0.05 mL of hot neutralized Ethyl alcohol and 2 mL of phenolphthalein indicator were added to the *SBO* sample, then titration with *NaOH* (N) normality until the appearance of the first permanent pink color, FFA calculated as shown:

1 $${\text{FFA as Oleic,}}\% = \frac{{{\text{ml of alkali}} \times {\text{N}} \times 28.2}}{{{\text{mass }}\,\,{\text{of }}\,\,{\text{test }}\,\,{\text{portion}}\,\,{\text{(g)}}}}$$

#### Moisture of gums measurements

The water content of gums is measured in the plant *QC* laboratory by the *AOCS* Official Method Ja 2b-87, Moisture in Gums, Karl Fischer Method^25^.

The weight of 0.5 ± 0.001 g of gums sample m in a dry titration vessel, then about 100–120 mL of (80:20 chloroform/methanol) added as a solvent, then a stirring by a stirring bar tell sample completely dissolved, then a titration with stirring against Karl Fischer reagent to the electrometric endpoint V_1_, Measure by the same procedure a blank test without gum sample and get endpoint V_o_.

Karl Fischer, reagent Standardization, is made by Placing a quantity of 80:20 chloroform/methanol in the titration vessel, adding a *V* ml of Karl Fischer reagent to give the endpoint, the weight of 150–350 mg of sodium tartrate w, (*Na*_*2*_*C*_*4*_*H*_*4*_*O*_*6*_*, 2H*_*2*_*O*) then titration to reach the endpoint, The Karl Fischer Reagent Factor *F*, in milligrams of *H*_*2*_*O* per ml of reagent, is calculated according to the formula *0.1566* × *w/v* for each mg of sodium tartrate is equivalent to:

2 $${\text{F}} = \frac{{2{\text{H}}_{2} {\text{O}}}}{{{\text{Na}}_{2} {\text{C}}_{4} {\text{H}}_{4} {\text{O}}_{6} . 2{\text{H}}_{2} {\text{O}}}} = \frac{36.04 }{{230.08}} = 0.155\,{\text{mg}}\,\,{\text{of}}\,\,{\text{water}}$$

where *F* is Karl Fischer Reagent factor in mg/ml^26^. The water content of the test sample is given as shown according to the following equation:

3 $$\% {\text{H}}_{2} {\text{O}},\left( {\frac{{\text{w}}}{{\text{w}}}} \right) = \frac{{\left( {{\text{V}}_{{\text{o}}} - {\text{V}}_{1} } \right) \times {\text{F}} \times 100}}{{{\text{m}} \times 1000}}$$

#### Hexane-insoluble matter HI %(impurities) of gums measurements

Solid impurities in separated gums are insoluble, such as hexane-insoluble matter *HI*%. It was calculated as in the plant *QC* laboratory by the *AOCS* Official Method Ja 3-87 by replicates two times for each test^27^.

The weight of 10 ± 1 g gums 250 mL flask, the addition of 100 mL Hexane and by shaking until dissolves, filter through filter funnel Corning *C* porosity, wash twice with 25 mL Hexane, Place funnel in the oven for 1 h at 105 ± 2 °C, then weighing, The HI% calculated as follow:

4 $${\text{Hexane}}\,\,{\text{insoluble}}\,\,{\text{matter}}\,\,{\text{HI,}}\% = \frac{{{\text{gain}}\,\,{\text{in}}\,\,{\text{mass,}}\,\,{\text{g}}\,\,{\text{of}}\,\,{\text{funnel}}}}{{{\text{mass}}\,\,{\text{of }}\,\,{\text{test }}\,\,{\text{portion}}\,{\text{(g)}}}} \times 100$$

#### Acetone insoluble (AI) measurements

Acetone Insoluble *AI* % of separated gums presents the pure *PL*. It was measured in the plant *QC* laboratory by the *AOCS* Official Method Ja 4-46 by Replicates 2 times for each test^28^**.** The weight of 1.9–2 (± 0.0005) g gums and 15 ml acetone were introduced to a centrifuge tube; the mixture was a worm in a water bath until the gums dissolved completely. The tube was placed in an ice bath at (0–5 °C) for 15 min. The tube was centrifuged at 1900 ± 100 rpm for 5 min. Then, 15 ml acetone was added and chilled in an ice bath (0–5 °C) for 15 min, and the acetone was decanted. The centrifuge tube was centrifuged again at 1900 ± 100 rpm for 5 min, then the acetone was decanted, and the tube was dried in an oven at 105 ± 2 °C for 30–45 min then cooled in a desiccator then weighed immediately. The following equation calculates the *AI*%:

5 $${\text{Acetone}}\,{\text{insoluble}}\,\,{\text{matter}}\,\,{\text{AI,}}\% = \left[ {\frac{{{\text{mass,}}\,\,{\text{g }}\,\,{\text{of }}\,\,{\text{residue}}}}{{{\text{mass, }}\,\,{\text{g }}\,\,{\text{of}}\,\,{\text{test}}\,\,{\text{portion}}}} \times 100} \right] - \% {\text{HI}},$$

#### Neutral oil loss contained in enzymatic degumming gums.

The neutral oil loss *NOL* % is the essential measure of degumming process efficiency^29^, which is trapped inside the separated gums that could be calculated from the analysis of *AI*% and moisture of gums by the following equation:

6 $${\text{NOL,}}\,\% = 100 - {\text{AI}}\% - \left( {{\text{moisture}}\,\,{\text{of }}\,\,{\text{gums}}} \right)$$

#### Neutral oil loss contained in soap stock

This analysis determines the total *NOL* in the soap stock test sample measured in the plant *QC* laboratory by the *AOCS* Official Method G 5-40^30^.

A weight of 8–10 g soap stock sample in an extraction cylinder and 125 mL of 50% alcohol and 50 mL of petroleum ether were added and shaken until a homogenous mixture was formed. The mixture cooled to 20–25 °C, and then 10 mL of aqueous potassium hydroxide (*KOH*) and 25 mL of 50% alcohol were added, and the cylinder was agitated gently until thoroughly mixed. The cylinder was allowed to settle until two layers were wholly separated by siphoning the ether/oil layer into a 500 mL separatory funnel. The extraction step was repeated at least 4 times using 50 mL of petroleum ether for each extraction, combining all extracted portions, drying the etheric extract into a Soxhlet flask and evaporating in a water bath. The residue dried in the oven at 105 ± 2 °C for 30 min. The NOL % is calculated from the following equation:

7 $${\text{NOL}},{\text{\% }} = \frac{{{\text{mass}}\,{\text{of}}\,{\text{neutral}}\,{\text{oil}}}}{{{\text{mass}},\,{\text{g}}\,{\text{of}}\,{\text{test}}\,{\text{portion}}}} \times 100$$

#### Actual oil loss in chemical and enzymatic degumming by plant mass flowmeters of oil calculation

Mass flowmeters of oil and gums are an indirect way to evaluate the degumming performance by knowing the total loss calculations for the reduction of the total amount of separated gums and measuring the amount of inlet and outlet oil from the enzymatic process as the following equation:

8 $${\text{Actual}}\,{\text{oil}}\,{\text{low}} = \frac{{{\text{inlet}}\,{\text{oil}}\left( T \right) - {\text{outlet}}\,{\text{oil}}\,\left( T \right)}}{{{\text{inlet}}\,{\text{oil}}\left( T \right) }} \times 100$$

### Plant processing methodology

#### Chemical refining method

Large plant-scale processing of *SBO* chemical refining runs at 600 tons/day. The hot oil conveyed for phosphoric acid is an 85% dosage used for acid conditioning. The oil conveyed to the oil/acid mixture was pumped into the reaction tank with a certain holding time of about 15 min, and then the oil cooled to 40–45 °C. The caustic soda added, which was calculated stoichiometrically, plus an excess of soda is required, which depends on the oil quality of *SBO* to acidified oil before a second mixer for neutralizing *FFA*. The 40–50% soda concentration must be diluted by demineralized water to a concentration suitable for the 7–14% process and depends on *SBO* quality.

**Figure 2:**
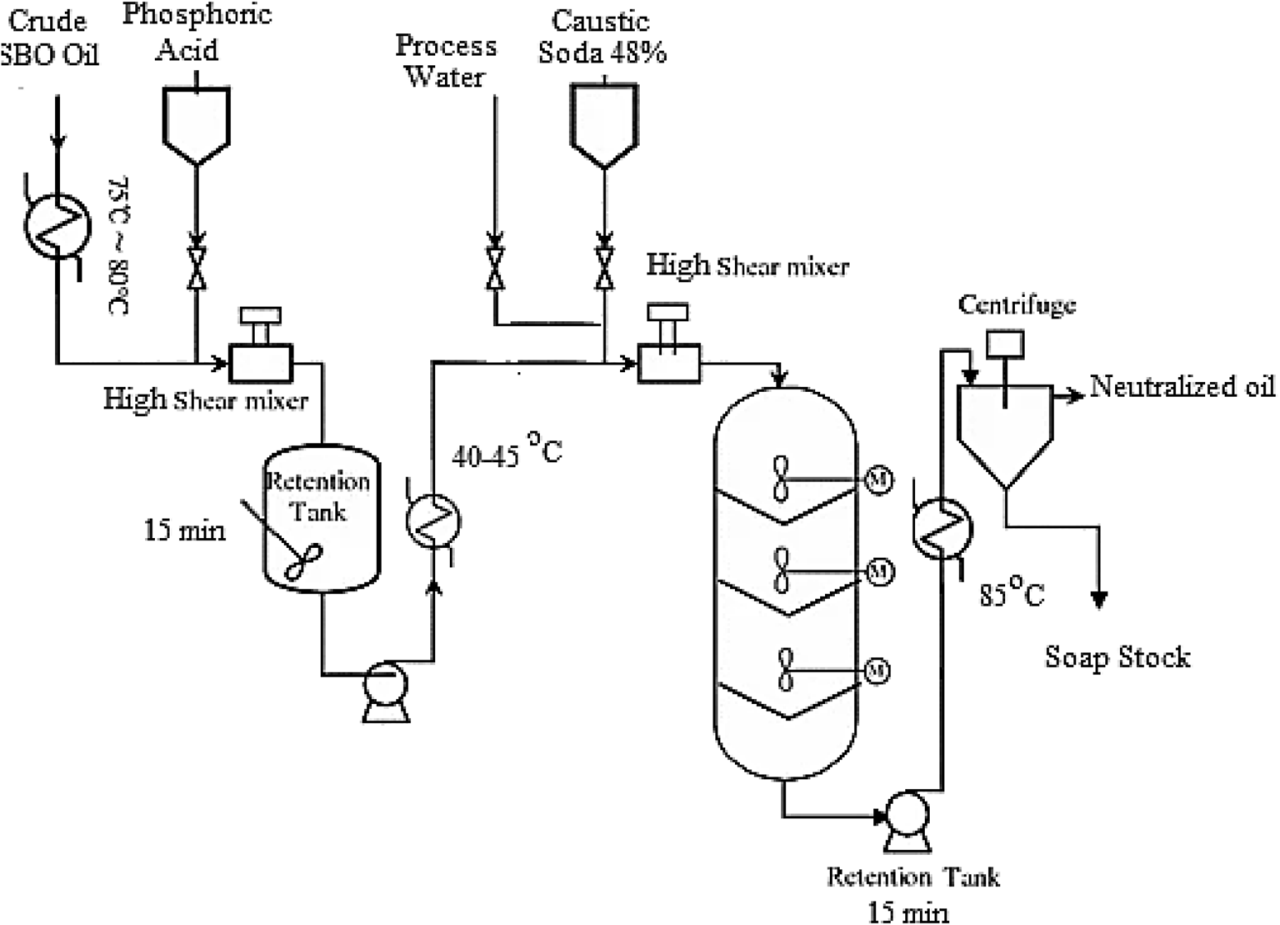
Standard (conventional) edible oil refinery.

After a strong mixing of oil and caustic soda in a mixer, the mixture is conveyed to the hydration tank for 20 min, called long-mix neutralization with agitation, then to the first centrifuge separator, separating the soapstock from the oil mixture. The outgoing neutralized oil flow rate is monitored with a flowmeter to calculate the *NOL* %. The soap stock is fed into the soapstock tank down the separator and conveyed with a specific pump for further processing. Acidified wash water is used to lower the soap contents in the neutral oil. For this purpose, citric acid must be used, a small amount of the citric acid solution as a function of the water flow rate. The oil is conveyed to the mixer to ensure intensive mixing of oil, and it is acidified and washed with water. It is then in the separator funnel, where the soap separates and goes to the soap stock tank. The washed neutralized oil was bleached with a bleaching agent. The bleached oil was subjected to the deodorization step. The flow diagram of the neutralization process is cleared in Fig. 2^31^.

Oil yield considerations for neutralization processes in long-mix neutralization refining losses^32^. It was monitored in several ways to determine refining losses^33^. The *NOL* % method has been supplanted by the *NOL* Method *AOCS* Official Method G 5-40. The process determines the weight of the neutral oil, consisting of triglycerides and the un-saponifiable components, which refers to the nonpolar components in *SBO*, in an *SBO* oil sample minus the more polar compounds such as *FFA* and PL that are targets for removal in the refining process. The neutral oil then becomes the theoretical amount, and the difference between it and the actual amount of *SBO* oil coming from refining gives a measure of the plant efficiency, as shown in Eq. (8).

### Quara enzymatic degumming

#### Acid conditioning

The large-plant scale processing of *SBO* enzymatic degumming runs at the 600 tons/day line. The acid step aims to get reaction medium pH at 3.8–4.2 for optimum enzyme activity. The acid step is done by mixing 85% phosphoric acid with the oil in a concentrated acid solution using a high-shear mixer. The reaction is done in a stirred tank reactor at 70–75 °C for 30 min.

#### Enzymatic reaction

**Figure 3:**
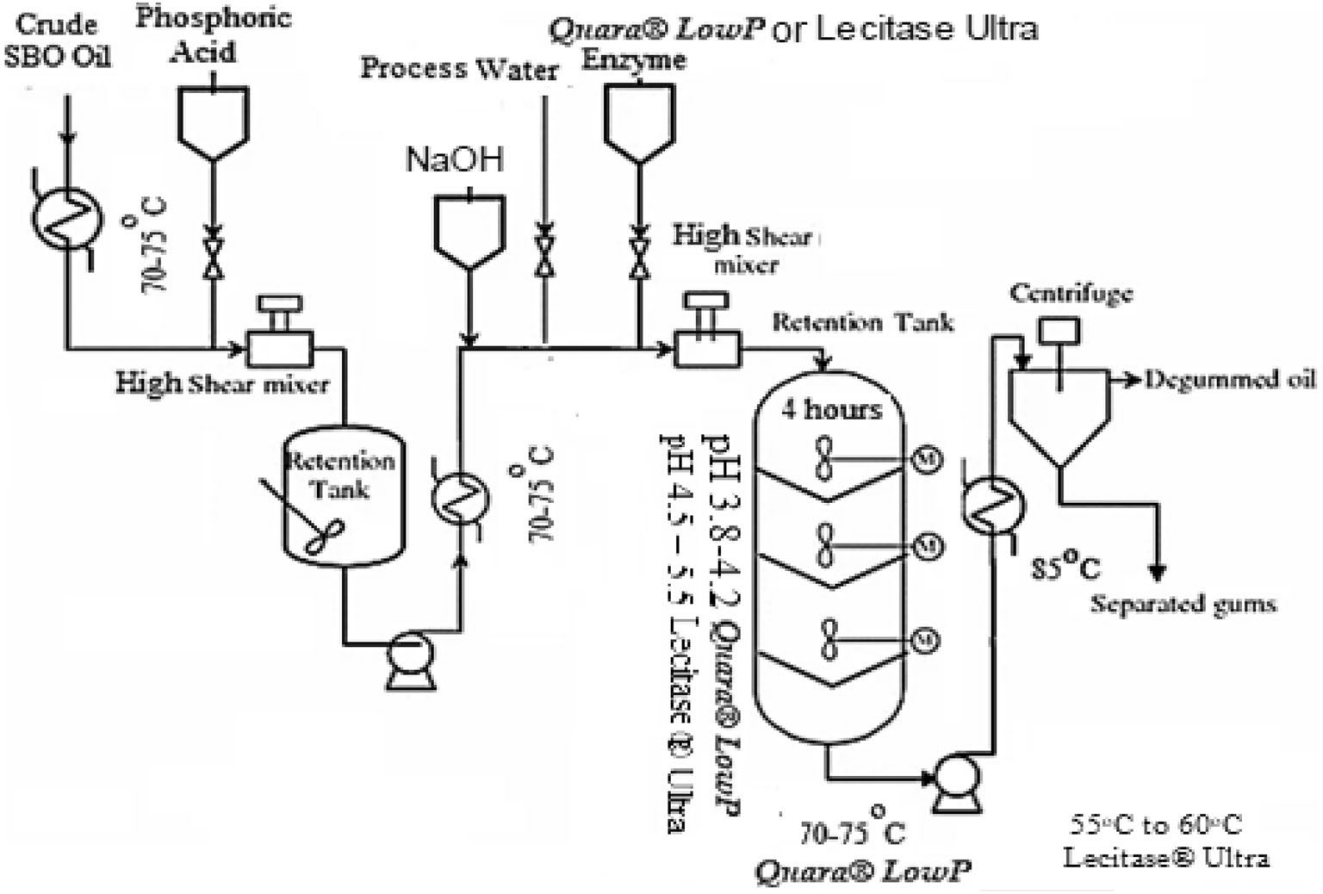
Quara low P and lecitase ultra enzymatic degumming flow diagram.

After acid conditioning, the water, Quara LowP, and Lecitase Ultra enzymes are added. The total water content in the reactor is 5% w/w of the oil. After the enzymatic reaction, which is 4 h in the enzymatic reactor, the oil mixture with enzyme and water is heated to 70–85 °C before being introduced to centrifuge. The gum was separated continuously by one self-cleaning centrifuge made by Westfalia^17^. The flowsheet in Fig. 3^34^ may considered in three sections: Acid conditioning, enzyme reaction, and gum separation.

Oil yield considerations for Quara LowP and LecitaseUltra enzymatic degumming of *SBO* monitored by *FFA* analysis where the *FFA* produced from hydrolysis of *PL* can be followed by analysis method AOCS official method Ca 5a‐40. The FFA increase can be estimated depending on the quantity of phospholipids in the oil. The expected FFA increase in the treated oil can be calculated from the original amount of phospholipids present in the crude multiplied by the ratio of molecular weights of FFA and phospholipids, approximately 282/750. Experimentally, half of FFA remains in the SBO. We typically get 65–75% of FFA released from PL.

We must note that not all the *FFA* remains with the oil phase after centrifugation. Usually, a significant amount of *FFA* is collected in the gum phase, so we measure the *FFA* before centrifugation.

Also, the residual oil in separated gums indicated by *AI*% measurement by *AOCS* Official Method Ja 4-46 method and moisture of separated gums by *AOCS* Official Method Ja 2b-8 method is an indication of an increase the oil yield compared with *NOL* in soap stock in the chemical refining.

## Results and discussion

The increase in yield evaluates the advantage of enzyme treatment over chemical refining from the Quara LowP and Lecitase Ultra enzymatic degumming of the fatty acid composition *FFA* and the reduction of the oil contained within the gum fraction, mainly composed of *LPL*. This oil inside the gums has been estimated from laboratory experiments. We also focused on forming *FFA* due to converting *PL* to *LPL*.

### Physicochemical analysis results

The Enzymatic and chemical degumming conditions and analysis results for different origins (American, Argentina, Ukraine, and Uruguay) of *SBO* which lead to P ≤ 10 mg/kg, shown in Tables S1, S2, S3, S4 in the supplementary file for Quara LowP enzymatic degumming, respectively. But Tables S5 and S6 in the supplementary file are specially for Lecitase Ultra enzymatic degumming of American, Ukraine *SBO*.

We analyze *FFA*%, *P* mg/kg, *Ca* mg/kg, *Mg* mg/kg, and *Fe* mg/kg for *SBO* for the four different origins. We listed out Phosphoric Acid dose mg/kg in both chemical and Quara LowP and *Lecitase Ultra* enzymatic degumming modes to see the phosphoric Acid 85% effect on the gums' pH. The Quara LowP and Lecitase Ultra doses in mg/kg were investigated to study the effect of enzyme dose on the enzymatic yield. Retention time for enzymes by hours, Quara LowP and Lecitase Ultra enzymatic water ratio % were also studied. Phosphorus in mg/kg and *FFA* for the produced enzymatic degummed oil was investigated to explain the increase in the *FFA* rather than the original *FFA* in SBO. The pH of gums *AI* % and moisture % of separated gums are also tabulated. The tables showed an increment in *FFA* and *NOS* in the separated gums of enzymatic degumming and the soap of chemical refining. The actual loss % per every 12 h in the plant running for chemical and enzymatic degumming was recorded.

### Parameters affect enzymatic degumming and chemical refining

#### Effect of chemical conditioning (CC)

A pretreatment step of the chemical refining and enzymatic processes is the addition of 85% phosphoric acid to achieve the hydratability of some *PL*. The acid step secures access to the phospholipids by chelating metal ions such as (*Ca, Mg* and *Fe*). It secures gums'' pH at 3.8–4.2 of Quara LowP enzymatic degumming and pH at 4.5–5.5 of Lecitase Ultra for optimum enzyme activity^35^. The acid step is done by mixing *SBO* with 85% phosphoric acid using a high-shear mixer.

As shown in Fig. S1, the Quara LowP enzymatic degumming performance was reduced at a pH lower than 3.8 and above 4.2 when combined with an operating temperature of 70 °C. The result at non-optimal conditions may include less *FFA* and *LPL* generation. The advantage of using Quara LowP compared with chemical degumming and Lecitase Ultra is that the caustic is not needed to raise the pH before Quara LowP enzyme addition, and the reaction temperature can be completed at 70 °C. in case of Lecitase Ultra the temperature must be between 55 and 60 °C, however, in chemical degumming it must be below 40–45 °C.

These features will lead to attractive advantages, such as yield improvement, energy saving, higher efficiency by reducing or eliminating the cooling step after acid treatment, and energy savings and reduced fouling of cooling heat exchanger^36^.

**Figure 4:**
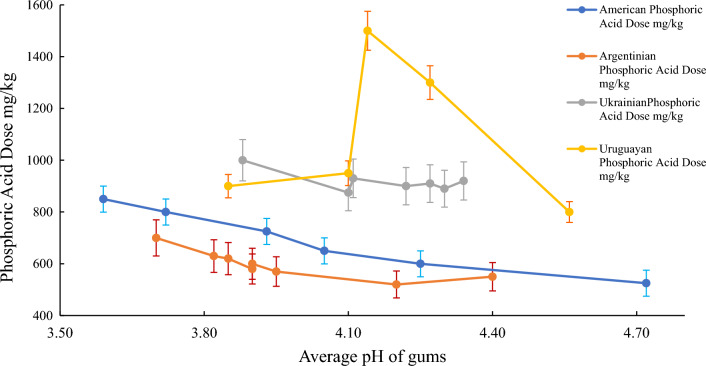
Acid dose effect on gums pH in quara low enzymatic degumming of different origins *SBO*.

Tables S1, S2, S3, S4 in supplementary file PLA reported the phosphoric acid dose optimization of American, Argentina, Ukraine and Uruguay *SBO*, respectively, the optimum phosphoric acid dose of about 600 mg/kg for American and Argentina *SBO* and about 900 mg/kg for Ukraine and Uruguay *SBO* to reduce the gums pH to 3.8- 4.2 as shown in Fig. 4 to show the best enzymatic activity and afforded the least loss % as illustrated in Tables S1, S2, S3, S4 in supplementary file *PLA* 23-09-2023. The acid dose will vary depending on feedstock quality, e.g., PL composition, to achieve the desired pH of 3.8–4.2. As the Novozymes manufacturer for Quara LowP and pH of 3.8–4.2 for Lecitase Ultra conditions recommended for the best activity^37^.

On the other hand, a high-shear mixer IKA is used to get a stable emulsion of enzyme and oil with a water phase^38^. The emulsion was maintained by agitation until the reaction was completed in about 4 h. At this point, the PL are divided into their corresponding LPL and fatty acids^5^.

The oil/water mixture is then heated to 85 °C and separated using a centrifuge. The pH of the water–oil matrix has been adjusted to be optimal, with pH = 3.8–4.2 of the Quara LowP and pH = 4.5–5.5 Lecitase Ultra supplied from Novozymes^18^. The enzymes react with the free form of (PC phosphatidylcholine, PI phosphatidylinositol, *PE* Phosphatidylethanolamine, and *PA* phosphatidic acid^39^, but the rate of the reaction varies from one phospholipid to another, for example, *Ca, Mg*, and *Fe* salts of *PA* are very slow and take from 4 to 6 h. After the reaction, the obtained oil with Quara LowP and Lecitase Ultra enzyme has low residual phosphorus < 10 mg/kg. The increment *FFA* in the treated oil measures enzymatic degumming process efficiency. It is calculated theoretically from the original quantity of *PL* present in the *SBO*, where the molecular weights of *FFA* and *PL* ratio are about 282/750. Half of the *FFA* stays in the oil.

9 $${\text{increase}}\,\,{\text{in}}\,\,{\text{ FFA}}\,\,{\text{\% }} = {\text{Phosphlipids}}\,\,{\text{\% }} \times \frac{282}{{750}} \times \frac{1}{2}$$

For example, *SBO* with 1.8% *PL* would increase *FFA* content by approximately (1.8% × 282/750 × 1/2) = 0.34%.

*PL* is almost totally converted into *LPL* by the *PLA* enzyme. However, the presence of *PA* and *PE* in the gums or the *PA* in the degummed oil indicates an incomplete reaction^40^. Separated gums obtained from the enzymatic reaction with *PLA 1* enzyme have low oil content (about 15%), and part of this oil is considered from half of the fatty acids produced by the reaction. The reduction of the amount of neutral fat in the gums^11^, the lower molecular weight of the *LPL* produced, and the *FFA* found in the gums measured in terms of acetone insoluble *AI%* in the dried gums (60–65%) is lower than that of water degumming (62–70%). However, it is paramount to note that the gum sample is visibly distinct, much less viscous, with a pale-yellow color, and the volume of the reacted gums is significantly reduced^41^.

#### Effect of the enzyme dose on the yield

A study of the impact of Quara Low enzyme dosage on degumming efficiency was conducted to investigate the best dose of the enzyme. The enzyme concentration was selected under the optimization of the loss%^42^.

**Figure 5:**
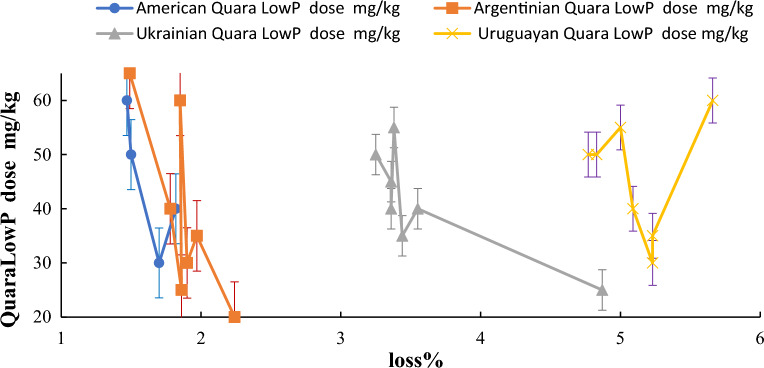
Dependence of the quara low P dose and loss % of different origins of *SBO.*

Figure 5 shows that the Quara LowP dose is very effective in the loss% of oil during the degumming process. The loss percentage is minimum to be 1.03 at 50 mg/kg. However, all doses gave the desired mg/kg values obtained P concentration from 5 to 10 mg/kg^43^.

#### Effect of the enzyme reaction time

**Figure 6:**
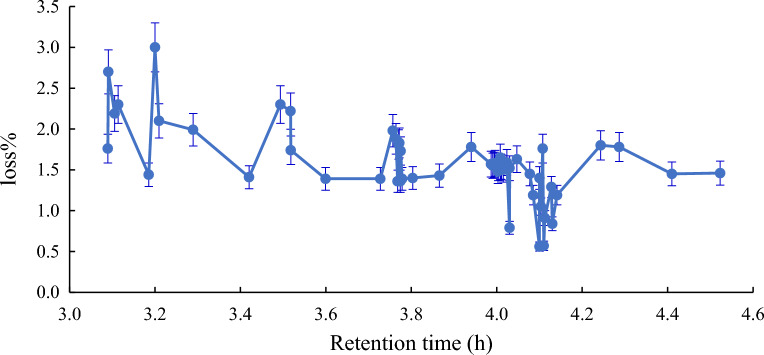
Retention time effect (h) on loss% in quara low P enzymatic degumming of American *SBO*.

**Figure 7:**
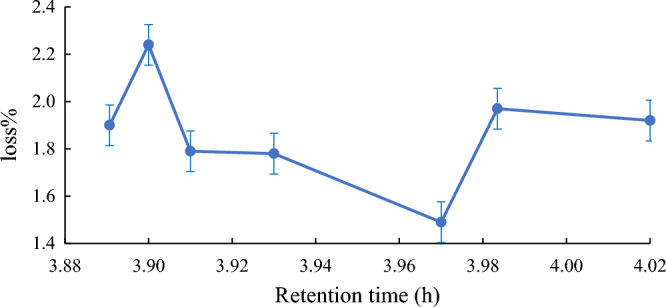
Retention time effect (h) on loss% in quara low P enzymatic degumming of Argentinian *SBO*.

**Figure 8:**
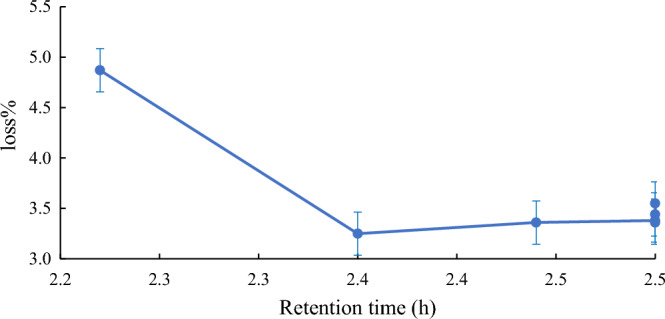
Retention time effect on loss% in quara low enzymatic degumming of Ukrainian *SBO*.

**Figure 9:**
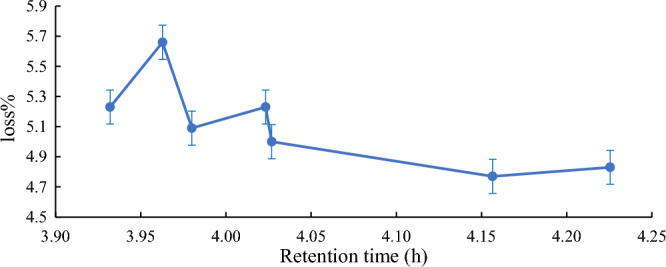
Retention time effect on loss% in quara low enzymatic degumming of Uruguay *SBO*.

The time of the Quara Low enzyme reaction of American *SBO* was also studied. Many retention times were investigated to get the best reaction time of the enzyme to afford the most negligible loss percentage. Figures 6, 7, 8 and 9 illustrate the optimum reaction time of the degumming process using Quara LowP. The results showed that the optimum time is 4 h to give more stable oil loss.

#### Effect of characteristics of the oil seeds

Table 3 reported the *SBO* characteristics before treatment according to their origins to show the effect of these limits on economic impact in terms of the Quara Low enzymatic degumming yield. The percentage of the inlet *FFA* was the lowest for the American seeds, with the highest for the Uruguay seeds.

**Table 3:** Oil seeds' characteristics, pretreatment, and quara low enzymatic degumming performance according to their origins.

Quality elements	American	Argentinian	Ukrainian	Uruguay
Inlet oil FFA	0.65	0.79	0.93	1.40
Inlet oil phosphorus, P mg/kg	877.71	821.88	798.96	762.4
Inlet calcium, Ca mg/kg	111.14	128.16	134.1	182.62
Inlet magnesium, Mg mg/kg	82.83	87.78	94.98	122.65
Inlet iron, Fe mg/kg	2.19	1.65	3.16	3.88
Total divalent metals, mg/kg	196.16	217.59	226.24	309.15
Final phosphorus mg/kg	6.66	6.86	10.61	12.32
Difference of in and out FFA (economic benefit)	0.48	0.37	0.48	0.33
Oil in gums % (economic benefit)	8.44	8.88	12.27	15.4
Oil in meal % (economic benefit)	1.24	1.43	1.58	1.83
Final FFA%	1.11	1.11	1.07	1.53

Table 4 reports the average loss% of treating the *SBO* with chemical refining and the average loss% of the Quara Low enzymatic degumming process. The Enzymatic yield is the subtraction of enzymatic degumming loss % from the chemical refining loss%.

10 $${\text{Enzymatic\,\,yield}} = \,{\text{Chemical\,refining\,loss\% }}\, - \,{\text{Enzymatic\,refining\,loss\,\,\% }}$$

**Table 4:** Oil seeds quara low enzymatic degumming yield.

Parameters	Seed Origin	American	Argentinian	Ukrainian	Uruguay
Plant data	Enzymatic oil loss %	1.58	1.87	3.60	5.12
	Chemical refining losses %	3.50	3.62	4.46	5.79
	Enzymatic yield % (economic impact)	1.92	1.75	0.86	0.67

**Figure 10:**
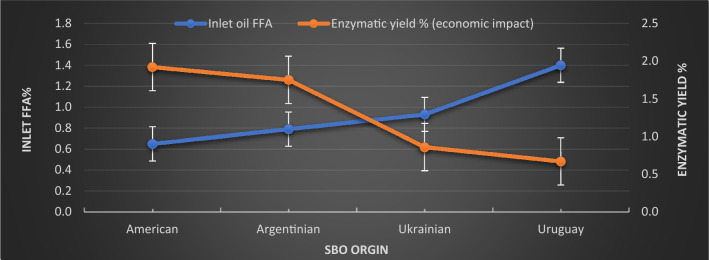
The dependence of the FFA of SBO and enzymatic yield of quara low enzymatic degumming of different origins of *SBO*.

Figure 10 shows the relationship between the Quara Low enzymatic degumming yield reported with inlet *FFA* of *SBO*. It is obvious that the economic impact (enzymatic yield) is inversely related to the percentage of the inlet-free fatty acid reported in Table 3. These findings could be attributed to the increment of the *PA* due to *PLD* enzymatic reaction inside soybean seed because of prolonged storage time with unacceptable conditions.

**Figure 11:**
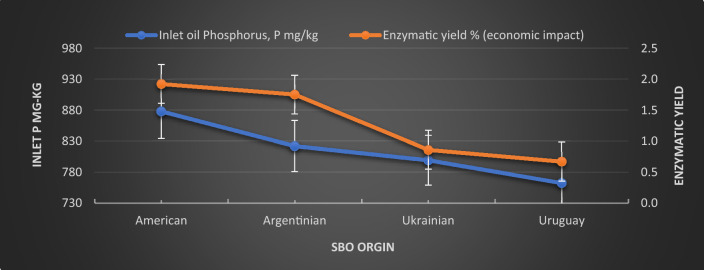
The relationship of the inlet phosphorus and the enzymatic yield of quara low enzymatic degumming of different origins of *SBO*.

On the other hand, the inlet phosphorus *P* in mg/kg could also affect the economic yield, as shown in Fig. 11, where the high inlet phosphorus concentration indicates a high phospholipid content. The high *PL* will result in a high yield because the *FFA* generated is considered an essential part of the Quara Low enzymatic degumming yield. The enzymatic yield is directly proportional to the inlet phosphorus *P* in mg/kg.

Theoretically, the *FFA* generated is half of the percent of *PL* in the *SBO* multiplied by the ratio of molecular weights of *FFA* as oleic acid and *PL*, 282/750, an indication shown in Eq. (10). For example, a *SBO* with 2% *PL* increases *FFA* content by approximately 0.38% according to the equation (2% × 282/750 × 1/2).

**Figure 12:**
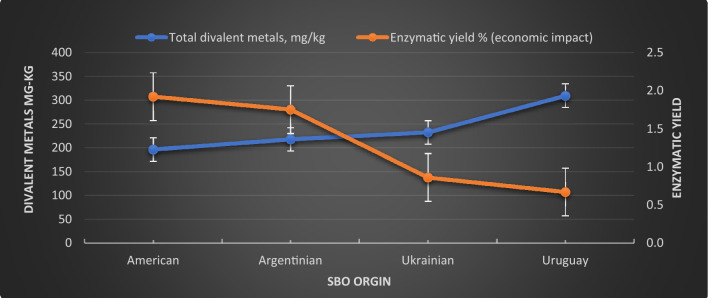
The enzymatic yield relationship with total divalent metals content in mg/kg of Quara Low enzymatic degumming of different origins of *SBO*.

Figure 12 illustrates the relationship between the Quara Low enzymatic degumming yield with total divalent metals, *Ca, Mg*, and *Fe* content in mg/kg. The high content of the divalent metals highly negatively affects the percentage yield of the enzymatic reaction because of its complexity with *PL*. This complex is difficult to convert into the free form of *PL*, which leads to a decrease in the efficiency of the degumming process with enzymes.

**Figure 13:**
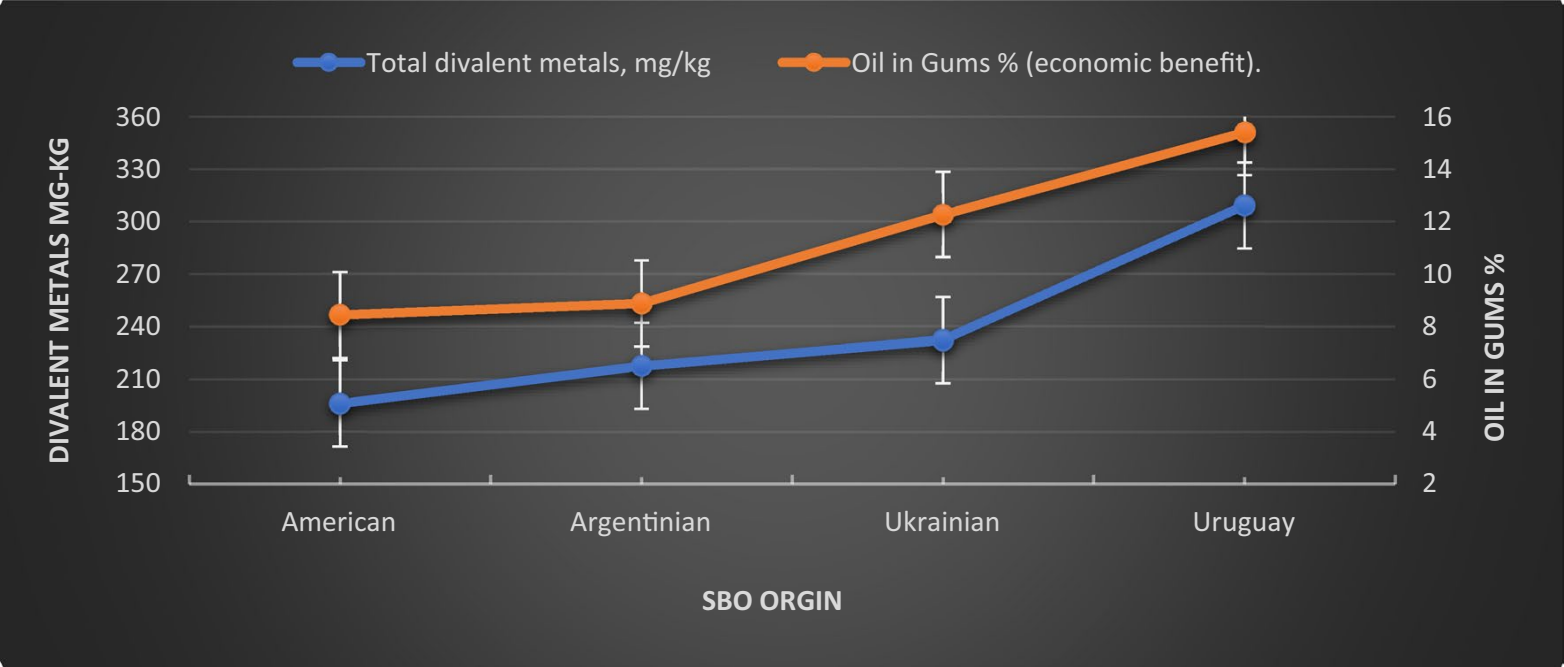
Neutral oil content in gums from different origins of *SBO.*

Figure 13 illustrates the neutral oil content in gums from different origins of *SBO*. It is clear that the range of the neutral oil content varies according to the quantity of the divalent metals. It has been reported that the NHP content present in *SBO* will be measured in terms of the amount of *Ca, Mg*, and *Fe*^7^. NHP is formed during harvesting, storing, handling, crushing, and extracting soybean seeds^44^.

Table 5 illustrates the degree of hydration of different types of *PL.* It is well known that the reason for the high level of *NHP* of the *PL* is the presence of the *PLD* enzyme inside the seeds. This enzyme makes the hydrolysis of all types of *PL* inside the seeds to afford *PA*^45^. The obtained free *PA* can form complexes with the divalent metals, forming water-insoluble salts. Consequently, the presence of divalent salts, especially the calcium one with *PI, PE*, and *PA,* will decrease the degree of *PL* hydration.

**Table 5:** The hydration of the different types of *PL*.

Phospholipid	PC	PI	PI (calcium salt)	PE	PE (calcium salt)	PA	PA (calcium salt)
Relative rate of hydration	100	44	24	16	0.9	8.5	0.6

Moreover, the obtained complexes will prohibit the effect of the manipulation of the changing of the pH, and this is because their hydration is independent of the pH value of the reaction mixture from Table 6. It can be seen that the Ca salts of the *PA* will have zero charges at all pH ranges.

**Table 6:** Charges of phosphatides with pH change.

pH	PC	PE	PI	PA	Ca-PA
2	+	+	0	0	0
3	( +)	( +)	(0)	(0)	0
4	( ±)	( ±)	(−)	(−)	0
5–7	±	±	−	−	0
8–9	±	±	−	(2−)	0
> 10	±	−	−	2−	0

( ) means the transition between the value at lower and higher pH.

Table 6 shows that the calcium, magnesium, or Iron salt of *PA (PA-Ca, Mg or Fe*) is free from the charge in all pH values because the divalent *Ca, Mg,* or *Fe* ions form salts with the two dissociated hydroxyl groups of the phosphate part. Consequently, metal salts of *PA* remain in water-degummed oil. They are the major constituents of nonhydratable phosphatides *NHP*.

The old chemical refining method was a caustic treatment for removing all *PL* and free fatty acids from oils. This step transforms the oil's *FFA* into the soap to help produce an emulsion to remove the NHP. The soap emulsion and hydratable PL trap massive oil in the separation step. As a result, this method is not recommended economically. It reacts with all oil components, such as *FFA*, triacylglycerols, diacylglycerols, monoacylglycerols, tocopherols, sterols, etc. The *NOL* in the separated gums^18^ and by this method, it is reported that gums removed in the chemical degumming process have 18–10% trapped oil.

On the other hand, our enzymatic degumming process is more economically recommended. As shown in the previously discussed, it is obvious that the quantity of free oil in the removed gums was in the range of 8–16%, and as mentioned, the divalent metals calcium, magnesium, and iron in the different origins of *SBO* oil make the enzymatic reaction less efficient.

#### The calculations' economic added value of the enzymatic degumming process

We can use the enzymatic yield gain to calculate the value added rather than the old chemical refining method, for example, the American *SOB.* We can calculate the cost benefits as shown in Table 7.

**Table 7:** Enzymatic value added by EGP.

	American SOB	
	Quara low enzymatic degumming	Lecitase ultra enzymatic degumming
1000 kg American SOB cost	EGP 60,000.00	EGP 60,000.00
1 kg enzyme cost	EGP 1200.00	EGP 1000.00
optimum enzyme dose g/Ton SOB	EGP 60.00	EGP 50.00
Enzyme per ton SOB Cost	EGP 72.00	EGP 50.00
Enzymatic yield gain %	1.92%	1.78%
Gain value add per 1 Ton *SOB*	EGP 1080.00	EGP 1018.00
Gain add value per day (600 T/day)	EGP 648,000.00	EGP 610,800.00
Gain value add month	EGP 19,440,000.00	EGP 18,324,000.00
Gain add value year	EGP 233,280,000.00	EGP 219,888,000.00

## Conclusion

Herein, the degumming process of the different origins of *SBO* has been discussed. The source of the oil seeds was used to illustrate the characteristics that could affect the oil losses during the degumming step. It has been noticed that the oil loss can be lowered according to the amount of *PL* present in the *SBO*. However, *SBO* processed by conventional degumming gives rise to losses that are always proportional to the amounts of *PL*. The increment of the amount of free fatty acids content after the enzymatic degumming process is considered a yield gain in the enzymatic degummed oil. The divalent metals content and *FFA* of the *SBO* have a negative effect on the yield gain of the Enzymatic degumming process. It was found that the origin of the oil highly affects the optimum dose of the enzyme used; however, the optimum reaction time of the degumming process using Quara LowP was found to be 4 h to give the more stable oil loss. Finally, the value added was calculated in terms of Egyptian pounds as a back investment, especially for the used enzyme in the degumming process.

In conclusion, the benefits of the enzymatic degumming process compared with the chemical one could be summarized as follows:

1. Higher yield.
2. There is less residual oil in the gum.
3. There is no oil loss in soap stock.
4. No wastewater.
5. No cost of effluent treatment.
6. No generation of soap stock is the more environmentally friendly process.
7. No low value of soap stock generation.
8. There is no capital cost of soap stock water treatment.
9. The enzyme is biodegradable with no residual enzyme activity in the final oil.

### Supplementary Information

Supplementary Figure 1.

Supplementary Tables.

## Data Availability

The data used and analyzed during the current study are available from the corresponding authors upon reasonable request.

## Abbreviations

AI: Acetone insoluble matter
AOCS: American oil chemists' society
CC: Chemical conditioning
FFA: Free fatty acids
HI: Hexane insoluble matters
HPL: Hydratable phospholipids
LPL: Lysophospholipids
NHP: Non-hydratable phospholipids
PA: Phosphatidic acid
PC: Phosphatidylcholine
PE: Phosphatidylethanolamine
PI: Phosphatidylinositol
PL: Phospholipids
PLA: Phospholipase A enzyme
PLC: Phospholipase C enzyme
PLD: Phospholipase D enzyme
QC: Quality control
