# Supplementary material for: Case study of chemical and enzymatic degumming processes in soybean oil production at an industrial plant
Source: Sci Rep. 2024 Feb 19;14:4064. doi: 10.1038/s41598-024-53865-9 (PMC10876682; doi:10.1038/s41598-024-53865-9)
Supplement: Supplementary file 1 — Supplementary Figure 1. [file 41598_2024_53865_MOESM1_ESM.docx]

**Industrial plant case study of chemical and enzymatic degumming process of soybean oil production**

*Maged Khamies ^1^, Mohamed Hagar ^1,2^, Taher S.E. Kassem ^1^, Amira H.E. Moustafa^1^**

**1** Chemistry Department, Faculty of Science, Alexandria University, P.O. 426 Ibrahemia, Alexandria 21321, Egypt.

**2** Faculty of Advanced Basic Sciences, Alamein International University, Alamein City, Matrouh Governorate, Egypt.


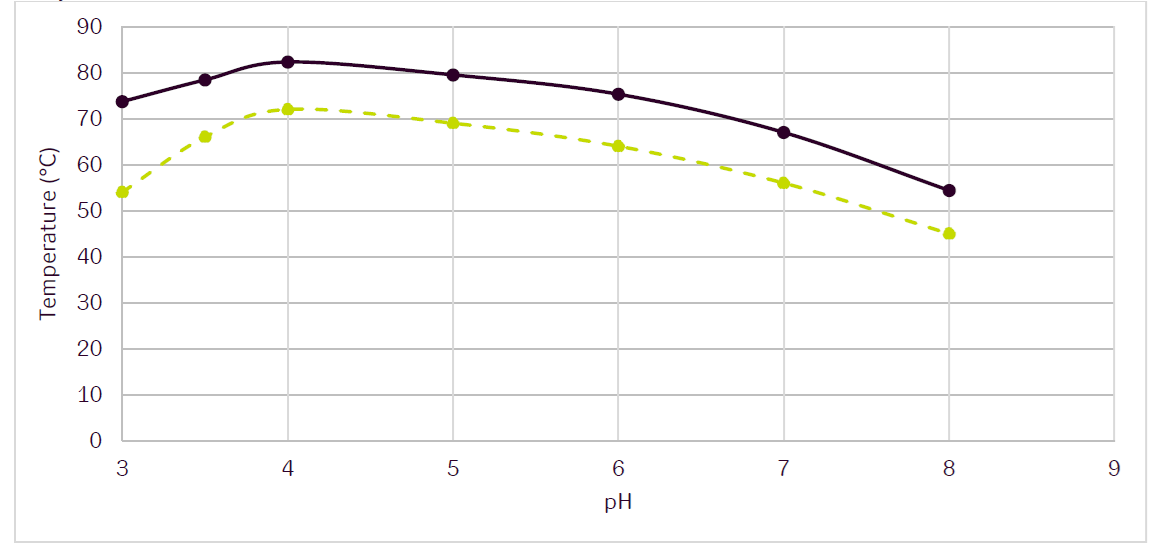


**Figure 1s.** Denaturation temperature as function of pH. The dotted line shows the start of denaturation and the solid line is the maximum denaturation rate [1].

**References:**

[1] Dijkstra, A. J. (2018). Enzymatic gum treatment. In Lipid modification by enzymes and engineered microbes (pp. 157-178). AOCS Press.
